# *¡Hola! Nice to Meet You*: Language Mixing and Biographical Information Processing

**DOI:** 10.3390/brainsci11060703

**Published:** 2021-05-26

**Authors:** Eneko Antón, Jon Andoni Duñabeitia

**Affiliations:** 1 Humanitate eta Hezkuntza Zientzien Fakultatea, Mondragon Unibertsitatea, 20500 Mondragon, Spain; eanton@mondragon.edu; 2 Centro de Investigación Nebrija en Cognición, Universidad Antonio de Nebrija, 28015 Madrid, Spain; 3 Department of Languages and Culture, The Arctic University of Norway, 9019 Tromsø, Norway

**Keywords:** language mixing, code-switching, multilingual learning, bilingual schooling

## Abstract

In bilingual communities, social interactions take place in both single- and mixed-language contexts. Some of the information shared in multilingual conversations, such as interlocutors’ personal information, is often required in consequent social encounters. In this study, we explored whether the autobiographical information provided in a single-language context is better remembered than in an equivalent mixed-language situation. More than 400 Basque-Spanish bilingual (pre) teenagers were presented with new persons who introduced themselves by either using only Spanish or only Basque, or by inter-sententially mixing both languages. Different memory measures were collected immediately after the initial exposure to the new pieces of information (immediate recall and recognition) and on the day after (delayed recall and recognition). In none of the time points was the information provided in a mixed-language fashion worse remembered than that provided in a strict one-language context. Interestingly, the variability across participants in their sociodemographic and linguistic variables had a negligible impact on the effects. These results are discussed considering their social and educational implications for bilingual communities.

## 1. Introduction

Social events are situations mostly defined by human interactions, and they often imply meeting new people. In a world that is steadily becoming less monolingual [1,2], these interactions may happen in any of the different languages known to the speakers, or even in a mixed-language fashion (as code-switching is a very common behavior, see [3,4,5,6]). In the current study, we explored whether the biographical information (i.e., relevant pieces of information regarding an individual’s life) shared in such a social interaction scenario is differently learned as a function of the languages used. Are we equally able to recall the features of our new acquaintances if the information we got from them was presented in one vs. two languages?

Questions such as this, which relate the knowledge and use of different languages with other cognitive abilities (e.g., memory), are of major relevance to understanding the extent to which domain-general cognitive processes and language interact with each other. Language is not only our most powerful tool of communication. It is also our main way of interacting with reality, and therefore variations in language settings or contexts could potentially cause variations in processes mainly guided by other high-order cognitive abilities. Along these lines, research over the last half-century has shown that language can filter how reality is perceived and understood (see the Sapir–Whorf hypothesis, [7], see also [8,9,10]), approached, and interacted with [11,12,13,14,15]. Furthermore, and more importantly for the purpose of this study, recent research suggests that language contexts mediate how the reality is categorized ([10,16,17,18,19,20,21,22], but see [23,24,25] for evidence putting into question the deterministic perspective of the Sapir–Whorf hypothesis).

The categorization of the reality surrounding us, and of its information, appears to be an evolutionary vital cognitive process that starts developing at a very young age [26] and that is present not only in humans but also in other species [27]. It is a basic cognitive ability that helps us make sense of what is around us, both consciously and unconsciously [28,29,30]. Crucially, categorization is an efficient way to learn and assimilate new information from the reality; potentially infinite individual encounters can be classified in a limited number of taxonomies that help us organize what we know and experience, and that allow for induction-based generalization [26]. It is important to note that exposure to and interaction with reality not only involves facing new general information, events, and elements, but also new persons. Social categorization plays a crucial role in social interactions, as it is the base of group-based evaluations (generally based on beliefs and stereotypes [31,32]), and it often happens spontaneously when we classify individuals based on the categories they belong to (most commonly age, race, and gender; see [33,34,35,36,37,38]). 

Critically, the language in which our interlocutors interact with us can also serve as a reality categorizer, given that social situations are often mediated by language. Inasmuch as the language/s of the speaker are not salient until she starts speaking, language-based social categorization has not received as much attention as other more salient factors (e.g., race or gender) until very recently. Nonetheless, this is becoming an increasingly relevant field of study, and the evidence gathered in the last decade convincingly demonstrates that the language a person speaks is a determinant factor used to categorize that person. Along these lines, it has been shown that language-based social categorization and preferences are present from a very young age; children of all ages preferentially attach with speakers of their native tongue, better accepting presents from them, and even preferring them as friends (see [39]). Interestingly, the categorizing power of language has not only been reported between languages (i.e., people who speak the same language tend to be classified as belonging to the same social group), but even within-language accent variations create social categories [40].

One of the intrinsic problems of studies comparing the effects of native and non-native languages, language variations, or accents, is that those factors usually correlate with highly relevant sociolinguistic differences [41]. Different languages or accents often co-occur with different cultures or countries of origin, and the impact of pure language factors in isolation could consequently be hard to detect. Indeed, extra linguistic cues, such as cultural factors and the physical appearance of the speaker, can trigger language selection [42]. To explore the true impact of language itself in social categorization without its co-occurring factors, scientists have started exploring different bilingual realities, especially those in which both languages are local. Studies with bilingual young adults from the Basque Country (where Basque and Spanish are co-official) have shed some light on this issue. Molnar et al. [43] presented participants with speakers that spoke either Basque or Spanish. After an initial exposure and familiarization phase, participants had to complete an audio-visual lexical decision task in which the previously presented speakers produced some words in either the language they were associated with during familiarization, or in the other one. Responses were faster when the speaker-language association matched that of the initial phase, indicating that participants had created links between speakers and languages and, arguably, social categories. Hence, even in contexts in which more than one native language is present (i.e., bilingual communities), social categorization based on the specific language that guides interactions emerges as a basic and essential communicative strategy that helps individuals anticipate and predict the linguistic context to foster a more effective communication [44,45].

One key aspect to keep in mind is that bilingual speakers in bilingual communities do not experience exclusively single-language context interactions with their interlocutors (namely, contexts in which only monolingual-like encounters take place). It is often the case that social interactions occur in a dual-language context (namely, contexts in which both languages are used interchangeably). Along these lines, recent research has also explored social categorization and anticipation or prediction processes regarding newly met interlocutors in such dual-language situations. In one of the first studies looking at this, Martin et al. [46] exposed bilingual participants to speakers who would speak only one language and to others who would produce utterances in two languages. When participants were presented again with the same speakers, brain potentials revealed that they could anticipate the language in which the monolingual speakers would speak before any word was produced, but this was not the case with the bilingual speakers. 

The relationship between categorization and memory has been extensively explored and established [47,48,49,50]. When the elements to be learned are part of a previously established closely related semantic category, these elements are incorporated into it (e.g., [51,52]). In contrast, and in the absence of close referents, the creation of categories is an efficient way to remember completely new pieces of information as we encounter them, by grouping them rather than storing them as individual entries [26]. From the data presented above, it seems that, in monolingual contexts, the speakers, and the information shared in these scenarios, are easier to predict—and thus categorize and memorize—than situations in which languages are intermixed. Consequently, one could tentatively predict that this could modulate the ease of remembering those scenarios and the pieces of information shared in those contexts. In other words, it could be hypothesized that the information provided by speakers who mix languages could be harder to remember, as compared to information provided in a single language, because the speakers in the former situation would be harder to predict and categorize. However, as will be reviewed below, recent data from studies exploring the impact of language mixing in learning seem to somewhat contradict these predictions.

Until very recently, alternating between languages has been actively discouraged in bilingual formal schooling, fearing a hypothetical—albeit unproven—negative impact of language mixing on learning. Instead, bilingual education has often been carried out as independent monolingual instances (also known as the “one subject-one language” rule, e.g., [53]). Indeed, language has been shown to be a crucial element during encoding and retrieval (see, among others, [54]). As an example of this, Marian and Fausey [55] showed that participants showed higher accuracy in memory tasks if the language of encoding and retrieval was kept the same, as compared to when they were different (see also [56] for additional evidence for language-dependent encoding). Together, these findings suggest that language is a factor that drives encoding processes, acting as a cue during retrieval, and, thus, manipulating the linguistic context could arguably modulate the integration in and retrieval from memory. If language is an important cue for memory, then it seems reasonable to assume that mixing languages during encoding could potentially hamper later retrieval.

Importantly, recent behavioral and electrophysiological studies addressing this approach have repeatedly shown that mixing languages when conveying new information does not negatively impact learning as compared to situations in which information is presented in a single language [57,58,59]. However, these findings speak of null differences when remembering the pieces of information in isolation, regardless of who the speaker was. Put differently, these studies have explored how language mixing could impact the learning of different pieces of information that are unrelated to the speakers (e.g., non-biographical information). However, in everyday life, we constantly associate information with people (personal information, opinions, ideas…), as they help us communicate with them and have a clearer idea of who those individuals are and what they are like. In consequence, who said something can be as important as the piece of information itself. In the current study, we posit a series of questions that closely map onto this issue: if the language in which we first meet a person is an important cue to categorize her and her features, and this cue is a crucial feature for encoding and later retrieval, does it matter whether we have this first interaction in a single- or a dual-language context? Are the features associated with this person easier to remember if the interaction with him or her was in a strict single-language (monolingual-like) fashion? And, importantly for the context of the current Special Issue, do individual differences in the demographic and linguistic profile modulate these effects?

With that purpose in mind, a large-scale study was conducted to test the effects of linguistic context and other sociolinguistic factors in associative memory. We tested more than 400 Basque-Spanish lifelong bilingual (pre) teenagers with clear-cut differences in the number of years mastering their languages. They were presented with cartoon avatars that could speak only in Spanish, only in Basque, or use inter-sentential language mixing. These avatars would introduce themselves by giving some personal information, such as their name, age, job, or favorite food. Participants would be later tested (immediately after learning and one day after) to see whether there were any differences in the recall and recognition of the features of each avatar depending on the language they used during exposure and the individual linguistic variations. 

## 2. Materials and Methods

***Participants.*** Four hundred seventeen children with a mean age of 12.40 years (SD = 1.89, range = 9–16) took part in this study. They were all students from a Basque-speaking school (*ikastola*) in the Basque Country at the moment of the experiment. They received formal schooling in Basque, and they were all Basque-Spanish bilinguals. They all learned Basque and Spanish before the age of 3 (mean age of acquisition of Basque = 0.89, SD = 1.05; mean age of acquisition of Spanish = 0.07, SD = 0.87). Every participant was perfectly fluent in both languages, and they used them in their daily lives in and out of school. They were rated as highly proficient in both languages by their parents, although there was still variability in their proficiency (mean reported Basque and Spanish level in a 1-to-10 scale was 7.97, SD = 1.13; and 9.20, SD = 0.87, respectively). All the participants’ parents gave informed signed consent before the experimental session according to the ethical commitments established by the Ethics Committee of the Universidad Antonio de Nebrija that approved the experiment and its protocols.

***Materials.*** Six cartoon-like 3D speaking avatars were created using the avatar generator app Veemee©. Half of them had prototypical male features and the other half had female features in the face, body, and hairstyle. All the rest of the visually identifiable features (namely, clothing, gesticulation, and background) remained identical across avatars. Six native bilingual Basque-Spanish speakers (three males and three females) recorded the auditory material, and each speaker was paired with one avatar. Every speaker recorded the same information, both in Basque and Spanish, always speaking in the first person: the avatar’s name, age, profession, favorite animal, and favorite food. Thus, the pieces of information were different for every avatar, and so were the voices they had. The avatar-language association was manipulated, as some avatars spoke in Spanish, some in Basque, and some used inter-sentential language switching (created by combining Basque and Spanish sentences from the same speaker; hereafter *mixed* condition). Three lists were created to counterbalance the language-avatar associations among participants. In each of the lists, every language context (Basque, Spanish, or mixed) was associated with one male and one female avatar. Table 1 shows the information that each of the avatars provided the participants with.

***Procedure.*** This experiment was conducted in a computer room during school hours. Participants belonging to the same school class completed the experiment at the same time (*n* < 30 per session). To assure privacy, each participant worked individually on his or her own computer with their own headphones. The experiment was conducted using LimeSurvey©, and all the instructions displayed on the screen were both in Basque and Spanish. The teacher assigned to each class made sure that participants paid attention to the experiment and that they fully understood the instructions prior to starting the experiment.

Participants were randomly assigned to one of the three lists when they entered the experiment. In the exposure phase, the 6 avatars were presented in random order, one after the other, in a form of a video clip with sound. Every avatar provided the participants with the same pieces of information (name, age, profession, favorite animal, and favorite food), but each did so in a different order. Every participant saw two avatars (a male and a female) speaking only in Spanish, two avatars speaking only in Basque, and two other avatars that alternated between languages inter-sententially. After each trial of the exposure phase (i.e., after each avatar had given all the corresponding information), participants were asked to type in the name, to guarantee that they were paying attention. Only 2 participants (0.39% of the sample) failed 2 out of 6 names, 55 failed one name (10.66% of the sample), and the rest all responded correctly. 

**Figure 1:**
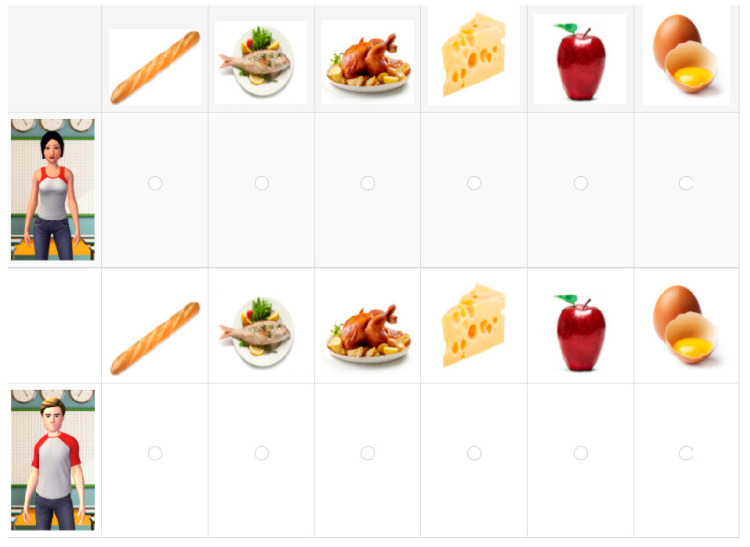
Example of the presentation of a male and a female avatar in the food assignation item.

Immediately after the exposure phase, participants went through a series of questions to measure their memory, some requiring them to freely recall information and some requiring them to recognize the correct features of the avatars. They first completed a recall test in which they were presented with static 2D pictures of the avatars, one by one in random order, and they were asked to type in each avatar’s name and age in blank squares underneath the picture. Once this task was completed for all the avatars, they completed a brief recognition test. Three multiple choice recognition trials were presented to the participants, one by one and in a random order: one related to the previously presented favorite food information, one related to the favorite animal information, and one related to the job of each avatar. For each of the three items, the screen set up was the same (see Figure 1).

Participants saw a 6 × 6 grid on the screen, on the left of which, and aligned with its rows, participants saw the pictures of each of the six avatars, in a random order across trials and participants. As a header in each of the rows, participants saw pictures depicting the six tokens of each category (i.e., 6 pictures of the food items mentioned by the avatars, 6 animals, or 6 pictures of the jobs), each picture aligned in one column. The task here was to assign each picture of food/animal/job to the avatar that previously mentioned it, by marking the intersections between the tokens and the avatars. Participants could only assign one element to each of the avatars, and all the avatars required a response. 

One day after, participants went back to the computer room in the same groups, and they repeated the free recall and recognition memory tasks. The items were presented in a random order across test days.

## 3. Results

**Figure 2:**
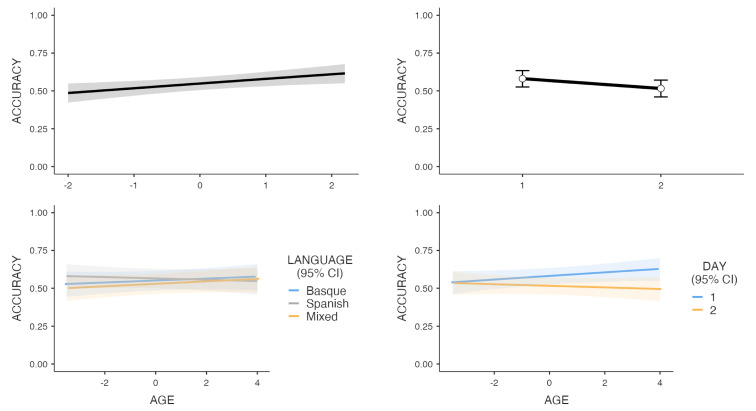
Graphical representation of the parameter estimates of the significant fixed effect Level of Basque (**upper left** plot) and Day (**upper right**), and the Language*Age (**lower left**) and Day*Age (**lower right**) interactions.

Participants’ responses in the memory tests were scored during the experiment for the recognition part, and offline coded for each item and each participant for the recall part. Table 2 shows overall mean correct responses in the free recall part (where name and age had to be retrieved) and in the recognition part (where participants needed to assign a profession, food, and animal to each avatar), grouped by language of exposition and split into the test day (Day 1 and Day 2; see also Figure 2).

Accuracy was coded as 0 (incorrect response) or 1 (correct response). Analyses were conducted with Generalized Linear Mixed-Effects Models using the *lme4* [60] package in R [61]. Prior to the initial construction of the maximal model, all numeric predictors were centered and scaled, and the categorical variables were coded using the deviation method. Significance *p*-values and Type II Wald Chi-Square (χ^2^) statistics for main effects, interactions, and planned comparisons were calculated using the *Anova* function of the *car* package [62]. All z-values higher than 1.96 were considered significant. Post-hoc and simple tests and the estimated marginal means (EMMs) for each specific factor combination in the significant interactions and the contrasts between them were computed using the *emmeans* package [63].

A maximal model was constructed, including in the fixed structure the factors Task (recall/recognition), Language (Spanish/Basque/mixed), Day (1/2), List (1/2/3), and the continuous variables Age (range: 9–17), Level of Spanish (range: 6–10), and Level of Basque (range: 6–10), together with all the potentially meaningful interactions between the factors. The model included random intercepts for participants and items and slopes for all within-item/participant predictors (a maximal structure; [64]). The model did not converge, and the random structure was simplified until convergence was reached. The final general model included random intercepts for participants and items. To find the best fitting fixed factor structure of the model, model comparison was done through a stepwise procedure. To this end, an automated model selection process was followed by sub-setting the maximum model using the *dredge* function from the MuMIn package [65]. In an iterative process, all the possible models with different fixed effect terms and interactions were contrasted and ranked according to their goodness-of-fit using the Akaike Information Criterion (AIC).

The simplest resulting model with the highest capacity to explain the accuracy data (*n* = 25,020 observations) included a fixed structure consisting of the factors Language, Day, Age, and Level of Basque, and the two-way interactions between Day and Age, and between Language and Age. This model was then analyzed in Jamovi [66] using the *GAMLj* module [67] (see Figure 2 for a graphical representation of the results). The main effect of Level of Basque was found to be significant (χ^2^(1) = 6.996, *p* = 0.008), with accuracy increasing as a function of the self-reported Basque proficiency level. The main effect of Day closely approached significance (χ^2^(1) = 3.368, *p* = 0.066), suggesting a drop in accuracy as a function of delayed testing. The rest of the main effects were negligible (all *p*s > 0.60). Importantly, the two interactions were significant. First, the interaction between Day and Age (χ^2^(1) = 21.598, *p* < 0.001) showed that accuracy increased with age on the immediate recall and recognition tests (Day 1), while it remained constant across ages on delayed tests (Day 2). Second, the interaction between Age and Language (χ^2^(2) = 8.869, *p* = 0.012) showed that, while performance was highly similar for Basque and Mixed conditions across ages (z = 1.169 and z = 1.795, respectively), older participants showed a drop in accuracy in the Spanish language context (z = 2.957). We interpret this finding as a consequence of the greater training in (or increased exposure to) language switching of older participants. Importantly, the simple tests between the different language contexts in the different age levels did not result as significant (all z < 1.1), suggesting that while the performance with the information processed only in Spanish varied with age, this did not yield differences between the language contexts.

## 4. Discussion

This study is, to the best of our knowledge, the first to explore the effects of language mixing in the memory for interlocutors’ biographical features or information. Participants were introduced to new persons who provided them with some personal information, closely resembling situations in which people meet for the first time. With just a single exposure to the avatars, participants remembered a significant amount of information, way above chance level, both right after the exposure phase and also one day after. Crucially, the data presented here clearly indicate that personal information conveyed in a strict one person-one language fashion was not better remembered than the same information provided in a language-switching scenario. When the individual differences in the linguistic profile of the bilingual children were considered, results demonstrated that their accuracy in both tasks increased as a function of their level of Basque (namely, better performance across languages when a higher Basque level was reported). More importantly, the results demonstrated that the age of the participants (as a proxy for their exposure to language switching environments) modulated their performance in immediate recall and recognition (but not in delayed tasks) and diminished the differences between languages.

The implications of this study are mostly twofold. Firstly, as this experiment resembled real-life situations in which new people meet for the first time, it could define the extent to which social categorization can follow language-based rules and how much this unconscious strategy can affect our daily interactions. Language mixing has been shown to make bilingual speakers’ languages less predictable during verbal exchanges (see [46]) and this, in turn, potentially makes the message less likely to be categorized. Language has also been shown to be an important cue for memory encoding and retrieval, and thus we expected the mixed condition to elicit worse performance as compared with single-language scenarios [54,56]). Following this rationale, we expected that information conveyed in a single-language context would be easier to categorize and encode together with the speaker, and consequently, easier to remember as compared to a mixed-language situation. We did not observe any differences in the memory performance of the children as a function of mixing languages when presenting the information. While a significant effect of age as a modulating factor of their performance with the information presented in Spanish was found, it is worth noting that no differences between the language contexts were found in the omnibus tests nor as a consequence of the simple tests derived from the interaction. This absence of differences could be potentially explained by the linguistic profile of our participants and their relation with the languages used in the experiment. As discussed in the Introduction, the creation of categories helps us assimilate elements and information together, and as a consequence, between-category differences are accentuated while within-category differences are more easily ignored [68]. Social categories follow the same rationale, and memory differences have been reported when comparing “own” to “other” social groups [34,36], including social groups corresponding to similar language variations or accents [41]. In the case of our participants, however, both languages used by the avatars were their “own” ones, and they do not necessarily imply differences in other social, cultural, or origin features (note that both languages were acquired before the age of 3). Furthermore, the speaking avatars did not show any prototypical Basque or Spanish-related visual feature or appearance that could trigger specific language preference or choice [42] because the physical details were kept neutral and similar across speakers. Even in the scenario in which the languages were mixed, participants most probably categorized the speakers as intra-group members, as switching is a behavior that bilingual speakers in bilingual communities often show spontaneously [3,69]. This could partially explain why recall and recognition processes for features presented in different linguistic contexts were unaffected by language mixing, both immediately after exposure and after a 24-h delay. Alternatively, it is worth discussing the possibility that, because both languages were present during the encoding phase, our participants could have been set in a “bilingual mode” [70,71]. In such language-switching context, participants could have experienced truly monolingual and bilingual interactions similarly, diluting any potential effect of a given language or language combination during encoding. However, considering the astonishing capacity that bilingual speakers set in multilingual contexts have shown to correctly categorize and anticipate the language/s their interlocutors speak [43,46], we deem this explanation highly improbable.

Secondly, the present findings add to and expand the previous evidence showing no detrimental effects of language mixing during learning across ages and contexts [57,58,59]. Language mixing has been actively avoided in formal bilingual schooling because it has been intuitively assumed that a switching context would harm learning. As a consequence of this scientifically ungrounded assumption, languages have been traditionally kept separated in learning contexts [72,73]. Recent evidence has shown that language mixing does not affect the understating, encoding, and future retrieval of conceptual information conveyed in a mixed language fashion [57,58,59], even if the underlying neural mechanisms involved in single- vs. dual-language exchanges are fundamentally different (see [74,75]). The present evidence generalizes previous evidence showing an absence of any detrimental impact of language mixing in conceptual learning and demonstrates that associative learning is not harmed by mixing languages either. Even though language switching has been repeatedly shown to induce some cognitive cost in experimental settings [76,77], the available educational evidence speaks for the full lack of detrimental effects of language alternation [78,79,80,81,82]. In the absence of negative evidence relating language mixing and learning, only the positive effects of freely using both languages in situations where they are contextually relevant and known to the speakers remain. Furthermore, the absence of negative consequences of using two separate codes might also speak in favor of theories that consider the bilingual linguistic repertoire as an integrated system rather than two separate ones [83,84,85].

Certainly, this is solely a first step in a long journey to our understanding of how variations in linguistic contexts and participants’ profile interact with each other during multilingual communication. Admittedly, the present findings can only be generalized to participants who are lifelong simultaneous and mostly balanced bilinguals. These kinds of bilinguals have been shown to be able to access translation equivalents at a minimal cost [86,87] and to spontaneously switch from one language to another [3,69]. The question remains of whether these effects would be replicated in a population with less mastery in at least one of the languages or with other language combinations. Importantly, future research should also bear in mind the different attitudes that individuals at test might have towards language mixing [88,89].

## Figures and Tables

**Table 1:** Basic information provided by each male and female avatar.

	Male1	Male2	Male3	Female1	Female2	Female3
**Name**	Aimar	Markel	Iker	Ane	Irati	June
**Age**	18	22	26	19	23	27
**Favorite Food**	Chicken	Apple	Fish	Bread	Egg	Cheese
**Favorite Animal**	Dog	Bear	Bird	Horse	Rabbit	Frog
**Profession**	Dancer	Cook	Photographer	Fire-fighter	Teacher	Athlete

**Table 2:** Grand averages per language of exposition of correctly remembered items (Recall test) and correctly identified items (Recognition test) as a function of test day (Day 1 and Day 2). Standard deviations are presented in parenthesis.

Language	Task	Day	Accuracy (% Hits)	
Basque	Recall	1	0.549	(0.498)
		2	0.502	(0.500)
	Recognition	1	0.554	(0.497)
		2	0.515	(0.500)
Mixed	Recall	1	0.556	(0.497)
		2	0.477	(0.500)
	Recognition	1	0.536	(0.499)
		2	0.490	(0.500)
Spanish	Recall	1	0.582	(0.493)
		2	0.493	(0.500)
	Recognition	1	0.559	(0.497)
		2	0.533	(0.499)

## Data Availability

The authors confirm that the data supporting the findings of this study are available within the article, and data involving individual participants can be obtained upon reasonable request.
